# Estimating the optimal dose of flupentixol decanoate in the maintenance treatment of schizophrenia—a systematic review of the literature

**DOI:** 10.1007/s00213-019-05311-2

**Published:** 2019-07-12

**Authors:** Loren Bailey, David Taylor

**Affiliations:** 1 Pharmacy Department, South London and Maudsley NHS Foundation Trust, Bethlem Royal Hospital, Monks Orchard Road, Beckenham, BR3 3BX UK; 2 Institute of Pharmaceutical Science, King’s College London, Fifth Floor, Franklin-Wilkins Building, 150 Stamford Street, London, SE1 9NH UK

**Keywords:** Flupentixol decanoate, Dose-response, Schizophrenia

## Abstract

**Rationale:**

The licensed dose range for the long-acting injectable antipsychotic flupentixol decanoate (Depixol®) in the treatment of schizophrenia is very broad. This provides little useful direction to prescribers and may ultimately result in patients receiving unnecessarily high doses.

**Objectives:**

We aimed to estimate the effect of dose of flupentixol decanoate on relapse rates in schizophrenia and on tolerability by expanding on an earlier review and including non-RCT and German-language studies, as well as using pharmacokinetic and pharmacodynamic data to offer guidance on dosing.

**Methods:**

A literature review using EMBASE, Medline, PsycINFO and PubMed was conducted. Treatment success rates at 6 months were extracted or extrapolated from the studies and plotted against dose to estimate a dose-response curve.

**Results:**

Data from 16 studies (*n* = 514) allowed estimation of a dose-response curve which rises steeply between the chosen placebo anchor (25% success rate) and 10 mg every 2 weeks before reaching a maximum between 20 and 40 mg every 2 weeks (80–95% success rates). Extrapyramidal side effects (EPSEs) were frequently seen (12–71% of participants) in that dose range. Two -weekly injections seem to provide the highest trough plasma concentration per dose administered and the lowest peak-to-trough concentration ratio. Plasma concentration varied up to 5-fold among individuals receiving the same dose.

**Conclusions:**

The optimal dose of flupentixol decanoate is likely to be between 20 mg and 40 mg every 2 weeks although higher doses may be required in some individuals owing to variation in drug handling. Doses of flupentixol should be individually established in the range of 10 to 40 mg every 2 weeks according to response and tolerability.

## Introduction

Flupentixol is a thioxanthine compound licensed for the treatment of schizophrenia and depression. An oral form of the drug was first marketed in Europe in 1965 by Lundbeck. The long-acting injectable formulation, flupentixol decanoate (Depixol®), followed in 1970. The usual effective dose range for the maintenance treatment of schizophrenia (as specified in the manufacturer’s formal summary of product characteristics) “lies between 50 mg every 4 weeks and 300 mg every 2 weeks, but some patients may require up to 400 mg weekly” (Lundbeck Limited 2017a). This represents a 32-fold difference between recommended minimum and maximum dose. The oral formulation has a much narrower recommended dose range of 3 to 18 mg per day—a 6-fold difference. The pharmacokinetic properties of the different formulations are summarised in Box 1 (Lundbeck Limited 2017b; Taylor et al. 2018), but offer no explanation for the wider dose range of flupentixol decanoate. The very broad range of doses recommended for the depot form give little useful direction to prescribers, and the long-acting nature of the formulation makes dose titration very challenging because of the prolonged time to steady-state plasma concentrations.

Reed and Fanshawe (2011) conducted a review in an attempt to establish a clear dose-response relationship for flupentixol decanoate. They concluded that survival rates (that is the proportion of patients remaining in a study) were highest at a dose of around 50 to 60 mg every 4 weeks. The authors acknowledged that the included studies recruited small numbers and had unusually high survival rates for schizophrenia (above 90%), suggesting that caution was required when interpreting the results. In addition, the studies reported only average doses and outcomes for groups as a whole, limiting the ability to draw conclusions for individual patients. Included studies were of different lengths (24 weeks to 18 months), and the survival rates used to plot the dose-response curve were taken from the end point of the study (i.e. they were not standardised to a specific time period).

In this current review, we aimed to estimate the optimal dose of flupentixol decanoate in the maintenance treatment of schizophrenia and to establish if the evidence from the literature supports the wide dose range specified by the manufacturer. We decided to extend the earlier work by Reed and Fanshawe by including non-randomised and observational studies (dose finding is not adversely affected by the absence of randomisation or control treatments), and those in the German-language literature. We also aimed to retrieve pharmacokinetic and pharmacodynamic data to provide the theory underpinning any dose recommendations, for example, plasma levels at the minimum effective dose or the relationship between flupentixol dose and dopamine D2 receptor occupancy.

**Table:** Box 1 Pharmacokinetic data oral vs injection

	Oral flupentixol dihydrochloride (Depixol®)	Intramuscular flupentixol decanoate (Depixol®)
Licensed dose range	3–9 mg twice daily	50 mg every 4 weeks to 400 mg every week
Bioavailability	40–55%	100%
Time to peak levels	4 h	4–7 days
Mean plasma half-life (SD)	35 h	17 days (9.2)
Time to steady state	7 days	2–3 months

## Method

In January 2018, a literature review was conducted. EMBASE, Medline, PsycINFO, and PubMed were searched using the terms flupentixol or flupentixol decanoate. Subheadings used were ‘adverse drug reaction’, ‘drug dose’, ‘drug therapy’ and ‘pharmacokinetics’ in EMBASE and ‘administration and dosage’, ‘adverse effects’, ‘pharmacokinetics’ and ‘therapeutic use’ in Medline. Additional studies were identified from references. Relevant studies had to include patients with schizophrenia or schizoaffective disorder, use the long-acting injectable (LAI) form of flupentixol, be at least 24 weeks in length, and include information on doses used and on relapse or deterioration in mental state as defined by the study. Relevant pharmacokinetic and pharmacodynamic studies had to include information on plasma levels and other relevant data. The search was repeated in January 2019. The Cochrane Systematic Review (Mahapatra et al. 2014) was also reviewed for eligible studies.

Treatment failure (defined as relapse, hospitalisation or clinical deterioration) rates were standardised to 6 months. If 6-month outcome was not reported in the study, the rate was calculated assuming a linear relapse rate as described in Kane et al. (2012). Treatment success rate at 6 months (100—treatment failure rate as described above) was plotted against mean or fixed dose in milligrams per 2 weeks to estimate a dose-response curve.

## Results

**Fig. 1:**
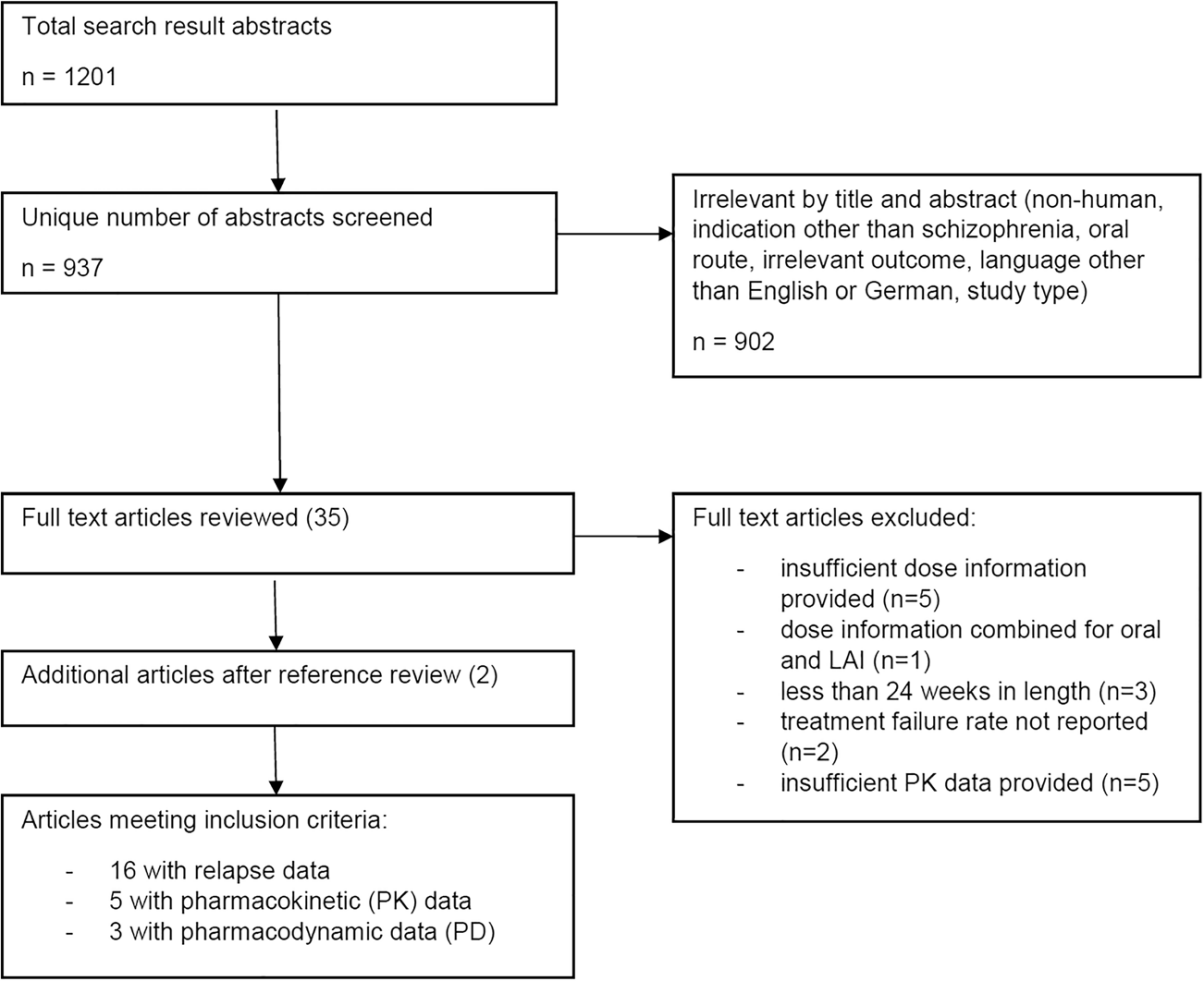
Study selection process

Sixteen studies meeting the inclusion criteria were identified, and eight studies reported on useful pharmacological data (see Fig. 1).

No additional studies of interest were found in the updated search.

The studies provided data on 21 different patient sets with a total of 514 patients and varied in duration from 6 months to 6 years. The study characteristics are displayed in Table 1. All studies included patients with schizophrenia, and the observational studies by Chiliza et al. (2016) and Shajahan et al. (2010) also included patients with schizophreniform disorder, persistent delusional disorder and schizoaffective disorder. Treatment failure was defined as relapse in nine studies (Chiliza et al. 2016; Cookson 1987; Gottfries and Green 1974; Johnson et al. 1987; Kelly et al. 1977; Knights et al. 1979; Laux et al. 2010; Pach et al. 1998; Wistedt 1981), deterioration according to global rating in one study (Agrup-Andersson et al. 1974), discontinuation due to poor response in two studies (Dencker et al. 1980; Shajahan et al. 2010), hospitalisation for psychiatric reasons in two studies (Knights et al. 1979; McCreadie et al. 1988), refusal to continue in two studies (Agrup-Andersson et al. 1974; McCreadie et al. 1988) and dropout from trial, not otherwise stated in three (Pinto et al. 1979; Steinert et al. 1986; Wistedt and Ranta 1983).

**Table 1:** Summary of relapse prevention studies of flupentixol decanoate (FD)

Study	Study design	Sample (*n*) duration	Stabilisation	Randomisation (*n*)	Other antipsychotics	Determinant of treatment failure
Agrup-Andersson et al. (1974)	RCT	Stable, female outpatients (56) 6 months	3 months—5 years	Continue dose (29) or halve dose (27)	Yes	Deterioration according to global rating or refusal to continue
Chiliza et al. (2016)	Open-label non-comparison	First episode, inpatient and outpatients (207) 12 months	During study period	N/A	No	Relapse in patients who initially responded
Cookson (1987)	RCT	Patients previously unresponsive to doses < 100 mg/2 weeks, improved on dose > 100 mg/2 weeks (18) 44 weeks	At least 3 months	Continue dose (9) or halve dose (9)	Yes	Relapse
Dencker et al. (1980)	RCT	Outpatients, previously stabilised on clopenthixol decanoate or flupentixol LAI (30) 12 months	None	Continue/switch to flupentixol palmitate (30)	No	Dropout due to unsatisfactory effect
Gottfries and Green (1974)	Follow-up/mirror image study	Outpatients, previously initiated on FD (58) up to 6 years	N/A	N/A	Not stated	Relapse
Johnson et al. (1987)	RCT	Outpatients, stabilised on FD < 40 mg/2 weeks (59) 12 months	At least 6 months	Continue dose (31) or halve dose (28)	No	Relapse
Kelly et al. (1977)	RCT	Outpatients stabilised on phenothiazine injections (15) 9 months	During first 9 weeks	FD 20 mg/3 weeks (15) or 40 mg/3 weeks (15)	No	Relapse or pregnancy
Knights et al. (1979)	RCT	Inpatients in acute relapse (28) 6 months	None	FD 40 mg/3 weeks (28)	No	Relapse or readmission to ward or day hospital
Laux et al. (2010)	Prospective cohort study	Inpatients and outpatients, initiated on FD in routine practice (94) 24 weeks	N/A	N/A	Yes	Relapse
McCreadie et al. (1988)	RCT—1-year follow-up	Patients responsive to oral treatment during acute first episode, subsequently randomised to FD (12) 12 months	Over 5 weeks	FD	Yes	Never discharged from hospital, re-admitted within 2 weeks of discharge, refusal of medication
Pach et al. (1998), Finkbeiner et al. (1998)	RCT	Outpatients who achieved remission within 3–12 weeks on FD dosed by treating psychiatrist (18) 12 months	3–12 weeks	FD 10 mg/2 weeks (19) or 20 mg/2 weeks (25) or flexible dosing (18)	Yes	Relapse
Pinto et al. (1979)	RCT	Outpatients stabilised on fluphenazine or FD (31) 18 months	At least 6 months	Continue/switch to FD	Yes	Dropout from trial
Shajahan et al. (2010)	Retrospective record review	Newly initiated patients (43 initiated onto FD) mean 13.6 months	N/A	N/A	Yes	Discontinuation due to inefficacy
Steinert et al. (1986)	RCT	Inpatients, acutely symptomatic (16) 12 months	None	FD 20–40 mg every 2 weeks	No	Dropout from trial
Wistedt (1981), Wistedt et al. (1982)	RCT	Outpatients, stabilised on FD (16) 24 weeks	At least 3 months	Continue FD or discontinue FD	No	Relapse
Wistedt and Ranta (1983)	RCT	Patients who had relapsed after drug withdrawal (17) 100 weeks	None	FD (dose as per previous dose requirement)	Yes	Dropout from trial

One study (Dencker et al. 1980) used flupentixol palmitate which has a relative molecular mass (RMM) of 672.92 g/mol. We converted the doses from this study to flupentixol decanoate (RMM 588.82 g/mol) equivalents using a factor of 0.875.

### Dose-response curve

The mean doses of flupentixol decanoate ranged from 10 to 333 mg every 2 weeks and averaged 46.2 mg every 2 weeks (see Table 2). Treatment failure rates at exactly 6 months were reported in nine of the studies. For the seven other studies, 6-month rates were calculated from the data given using the method described. Treatment success rates standardised to 6 months (100—treatment failure rate as described above) ranged from 62.5 to 100% (mean 85.4%).

**Table 2:** Summary of study results

Study	Number	Doses used (ranges)	Mean (mg/2 weeks)	Antipsychotic use (%)	Treatment failure over study period—*n* (%)	Treatment success at 6 months (%)	EPSE (%)	Anticholinergic Rx (%)
Agrup-Andersson et al. (1974)	29	Mean 40.7 mg/2 weeks (10–60 mg)	40.7	68	2 (7)	93	41	
Agrup-Andersson et al. (1974)	27	Mean 40 mg/4 weeks (10–30 mg)	20		6 (22.2)	77.8	36	
Chiliza et al. (2016)	207	Mean 11. 6 mg/2 weeks (10–30 mg)	11.6	0	33 (19.4)	90.3^a^	33	33
Cookson (1987)	7	Mean 333 mg/2 weeks (100–800 mg)	333	not stated	1 (14.3)	91.5^a^		
Cookson (1987)	9	Mean 118 mg/2 weeks (50-400 mg)	118	not stated	3 (33.3)	80.3^a^		
Dencker et al. (1980)	30	Mean 140 mg/4 weeks^b^ (not stated)	70	0	4 (13.3)	86.7		
Gottfries and Green (1974)	58	Mode 40 mg/2 weeks (20–60 mg)	40	not stated	36 (62)	92.7		
Johnson et al. (1987)	31	Mean 9 mg/week (4–20 mg)	18	0	2 (6.5)	93.5		
Johnson et al. (1987)	28	Mean 6 mg/week (1.7–10 mg)	12	0	5 (17.8)	82.2		
Kelly et al. (1977)	15	40 mg every 3 weeks	26.66	0	1 (6.7)	95.3^a^		
Kelly et al. (1977)	15	20 mg every 3 weeks	13.33	0	3 (20)	86.7^a^		
Knights et al. (1979)	28	40 mg every 3 weeks	26.66	0	10 (35.7)	64.3	71	43
Laux et al. (2010)	94		36.4	45	18 (19.1)	80.9		
McCreadie et al. (1988)	12	Mean 50 mg/3 weeks (50–100 mg)	33.33	not stated	3 (25)	87.5^a^		
Pach et al. (1998), Finkbeiner et al. (1998)	18	Mean 22.4 mg/2 weeks (5–40 mg/1–4 weeks)	22.4	38.9	3 (16.7)	91.4	22	28
Pach et al. (1998), Finkbeiner et al. (1998)	25	20 mg every 2 weeks	20	60	6 (24)	88	12	44
Pach et al. (1998), Finkbeiner et al. (1998)	19	10 mg every 2 weeks	10	42.1	6 (31.6)	74	26	53
Pinto et al. (1979)	31	Mean 36.6 mg/3 weeks (not stated)	24.4	3.2	0 (0)	100		42
Shajahan et al. (2010)	43	Median 60 mg/2 weeks (20–250 mg)	60	40	33 (76.7)	87.5		
Steinert et al. (1986)	16	Mode 40 mg/2 weeks (20–40 mg)	40	0	7 (44)	78^a^		
Wistedt (1981), Wistedt et al. (1982)	8	Mean 27.5 mg/3 weeks (10–40 mg)	18.34	0	3 (37.5)	62.5		
Wistedt (1981), Wistedt et al. (1982)	8	0 (Placebo)	0	0	6 (75)	25		
Wistedt and Ranta (1983)	17	Mean 31 mg/3 weeks (10–40 mg)	20.66	50	7 (43.8)	94.2		

*a Calculated

^b^Flupentixol palmitate doses have been converted to flupentixol decanoate equivalents

**Fig. 2:**
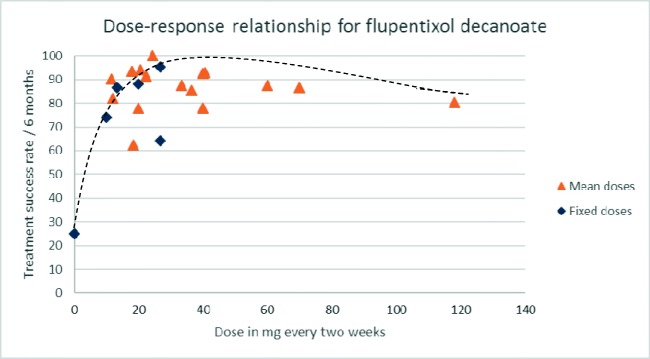
Dose-response relationship for flupentixol decanoate. Cookson (1987) dose of 333 mg/2 weeks (91.5% success) not included in graph

Treatment success rate at 6 months was plotted against mean or fixed dose in milligram per 2 weeks to estimate a dose-response curve (see Fig. 2). The placebo relapse data from Wistedt et al. (1982) was used as an anchor point in the dose-response curve plot.

When limiting the analysis only to studies who defined treatment failure as relapse (*n* = 530), the dose-response curve contains only two points beyond a dose of 40 mg every 2 weeks, but does retain the appearance of a flattening of effect between 20 and 40 mg every 2 weeks. Four studies (*n* = 74) included refusal to continue or drop out for any reason as a measure of treatment failure. All studies used doses of 40 mg every 2 weeks or less, and in one study (*n* = 31), no dropouts were reported. The dose-response curve retains its shape when these studies are excluded.

**Fig. 3:**
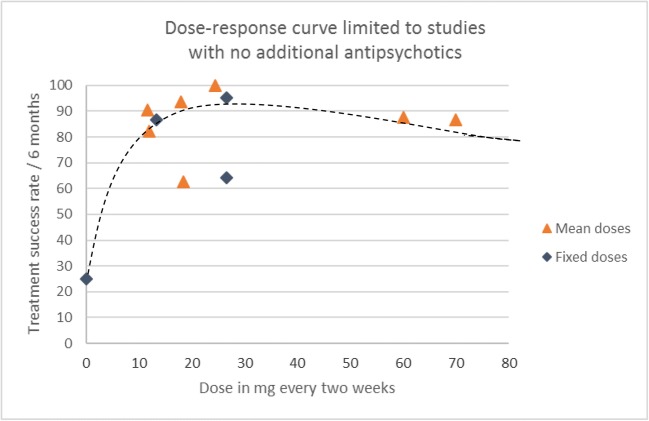
Dose-response relationship for flupentixol decanoate. Studies allowing additional antipsychotics have been excluded

Seven studies (*n* = 386) specifically stated that no additional antipsychotics were allowed during the study period. When limiting the analysis to only these studies, the dose-response curve shows a clear flattening of effect beyond a dose of 30 mg every 2 weeks (see Fig. 3).

One study (Kistrup et al. 1991) was specifically designed to establish the lowest effective dose for flupentixol decanoate in patients with schizophrenia. Patients stabilised on flupentixol decanoate had their dose systematically reduced by one quarter every 12 weeks until symptoms emerged. Eight of the 24 patients tolerated a dose reduction, and the average minimal effective dose was 60 mg every 2 weeks. Eleven patients remained stable on doses of 20 to 40 mg every 2 weeks; seven patients required doses of 50 to 80 mg every 2 weeks; five patients were treated with a dose of 100 mg every 2 weeks, and one person received 250 mg every 2 weeks.

### Tolerability

**Fig. 4:**
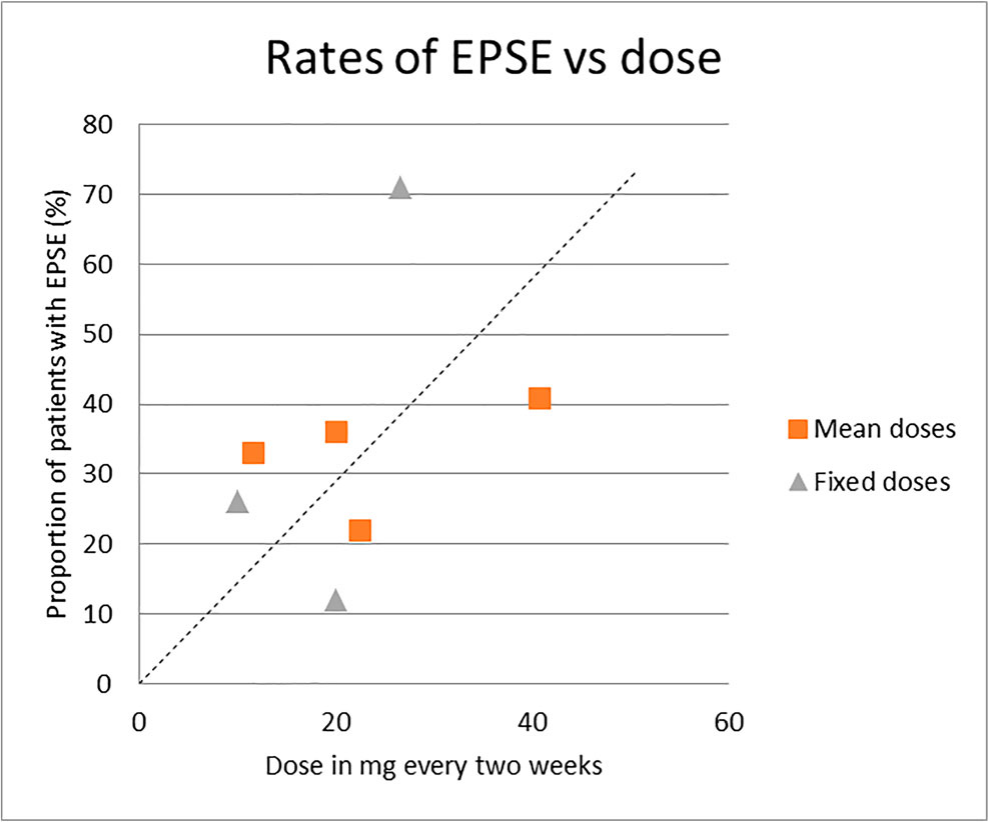
Rates of EPSE vs dose. The dotted line represents the line of best fit

**Fig. 5:**
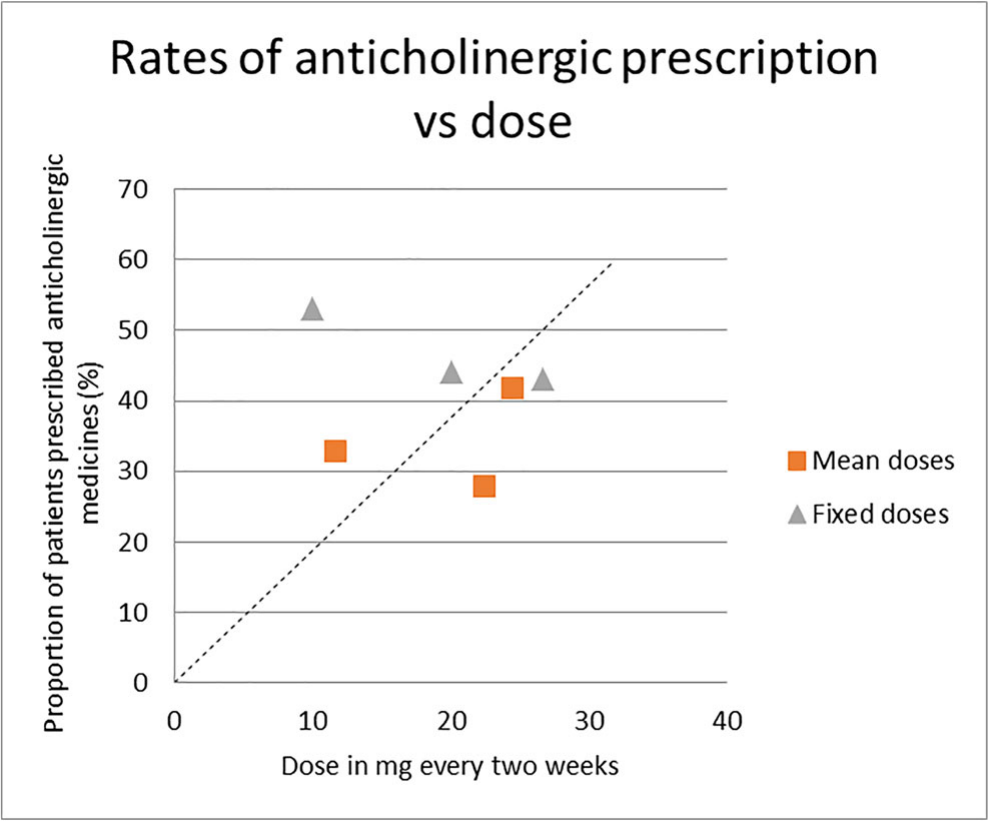
Rates of anticholinergic prescription vs dose. The dotted line represents the line of best fit

The proportion of participants experiencing extrapyramidal side effects (EPSEs) and the use of anticholinergic medication were reported in four studies each (see Table 2). We plotted rates of EPSE and rates of anticholinergic prescription against dose (see Figs. 4 and 5). There was no significant correlation between dose and rates of EPSE (*r* = 0.386; *p* = 0.96) or between dose and rates of anticholinergic prescription (*r* = − 0.227; *p* = 0.46). Extrapyramidal rating scale (EPRS) scores were recorded in two studies (Johnson et al. 1987; Kelly et al. 1977). In Johnson et al. (1987), EPRS scores declined in the half-dose group but remained constant in the full-dose group, and the authors noted a “significant fall in [tardive dyskinesia] during the 6 months following half-dose reduction (*p* < 0.05)”. The study by Kelly and co-workers (1977) found that mean adjusted EPRS scores were non-significantly lower in the 20 mg every 3 weeks group than in the 40 mg every 3 weeks group (0.32 and 1.44 respectively). Discontinuation owing to side effects was reported in five studies and ranged from 0% (Steinert et al. 1986) to 1% (Chiliza et al. 2016), 4% (Gottfries and Green 1974) and 25% (Laux et al. 2010) to 37% (Shajahan et al. 2010).

### Pharmacokinetics and pharmacodynamics

Pre-injection (‘trough’) flupentixol serum levels for individual subjects were reported in five studies (Cookson 1987; Jørgensen and Overø 1980; Kistrup et al. 1991; Saikia and Jørgensen 1983; Turbott et al. 1985), providing data for a total of 95 patients (Table 3).

**Table 3:** Summary of serum-level studies of flupentixol decanoate

Study	Assay method used	Treatment duration before level	Number	Mean dose in mg/week (range)	Injection interval (weeks)	Mean pre-dose serum level per mg/week in ng/ml (range)
Cookson (1987)	Radioimmunoassay (Jorgensen 1978)	> 4 weeks	18	112.75 (25–400)	2	0.26 (0.09–0.53)
Jørgensen and Overø (1980)	Radioimmunoassay (Jorgensen 1978)	> 2 months	21	112.7 (21.9–262.5)	4	0.11 (0.05–0.31)
Kistrup et al. (1991)	HPLC (Aaes-Jorgensen 1980; Larsen et al. 1985)	> 6 months	24	30 (10–125)	2	0.63 (0.21–1.8)
Saikia and Jørgensen (1983)	Radioimmunoassay (Jørgensen and Overø 1980)	> 3 months	23	16.5 (2.5–50)	2, 3 or 4	0.10 (0.01–0.26)
Turbott et al. (1985)	Radioreceptor assay-modified (Creese and Snyder 1977)	> 3 months	10	54.6 (6.67–140)	Not stated	0.34 (0.02–1.12)

The studies used different assay methods to estimate serum concentrations of cis-flupentixol with levels per dose administered ranging from 0.01 to 1.8 ng/ml per mg/week. The study by Kistrup et al. (1991), which used high-performance liquid chromatography, reported a higher average serum level per dose administered than the other studies (see Table 3). Jørgensen and Overø (1980) used flupentixol palmitate and normalised all serum levels to a dose of 100 mg every 4 weeks. In the study by Turbott et al. (1985), serum levels were expressed as haloperidol equivalents. Because of this heterogeneity in the assaying and reporting of serum levels, it was not sensible to consider these results together.

The study by Jørgensen et al. (1980) found serum levels to peak 7 days after the injection was administered, after which they declined with an average apparent half-life of 17 days. The ratio of peak to trough (pre-injection) serum levels varied from 1.6 to 10.4, a 6.5-fold variability in apparent half-lives between patients. Similar doses produced serum levels differing up to 5-fold for peak levels and 3-fold for trough levels. For example, in the 11 patients who received 100 mg every 4 weeks, trough serum levels varied from 1.1 to 3.0 ng/ml and peak levels varied from 2.9 to 14.7 ng/ml.

Saikia and Jørgensen (1983) also found an approximate 3-fold variation in the trough serum levels of patients receiving the same dose. For example, the five patients receiving 20 mg every 2 weeks had pre-injection serum levels ranging from 1.4 to 4.1 ng/ml. The average peak-to-trough level ratio was lowest in the two weekly group at 1.7, compared with 2.9 in the three weekly group and 2.6 in four weekly group. Variability of this ratio between patients was 2-fold in the two weekly group (*n* = 13), 3-fold in the three weekly group (*n* = 4) and 5-fold in the four weekly group (*n* = 6).

Three pharmacodynamic studies were identified (Balant-Gorgia et al. 1985; Farde et al. 1989; Reimold et al. 2007), all in patients taking oral flupentixol. A study by Balant-Gorgia et al. (1985) suggested that a plasma level of 2 ng/ml is required for clinical response (defined as 50% improvement in BPRS score over a minimum of 10 days). Doses ranged from 6 to 12 mg/day and steady-state plasma levels from 1.2 to 5.2 ng/ml. In a study by Reimold and co-workers (2007), therapeutic doses led to D2 receptor occupancies (D2-RO) between 50 and 70%, with a corresponding ED_50_ of 0.68 ng/ml. No patient’s plasma concentrations exceeded 2 ng/ml, and no D2-RO was higher than 70%.

## Discussion

### Dose-response curve

The dose-response curve rises steeply between the placebo anchor and 10 mg every 2 weeks before reaching a maximum between 20 and 40 mg every 2 weeks. At doses above 40 mg every 2 weeks, the curve begins to flatten and then fall, with higher doses seemingly producing lower treatment success rates.

For antipsychotics in general, the dose-response curve usually resembles an S shape (Davis and Chen 2004): at very low doses, response is minimal until a threshold dose is reached. Around this latter dose, the response curve rises steeply (almost vertically if not plotted logarithmically) before flattening off to a maximum, with dose increases producing no further effect. This characteristic pattern might be thought of as being analogous to a light switch—the drug is either ‘on’ of ‘off’ according to its dose. For long-acting injectable antipsychotics, this dose-response pattern has been demonstrated for haloperidol decanoate (Taylor 2005) with the dose of maximal effect estimated to be, at most, 100 mg every 4 weeks. It can also be seen with paliperidone palmitate and risperidone long-acting injections (Kane et al. 2003; Pandina et al. 2010).

The studies reviewed here are unable to clearly demonstrate a precise threshold dose of flupentixol decanoate—even doses of 10 mg every 2 weeks produced treatment success rates far exceeding those of placebo (although it should be noted that the position of the placebo anchor is derived from a single study). Other available estimates of the placebo effect are higher, e.g. 36% (Leucht et al. 2012) and 60% (Kane et al. 2012), but still lower than the effect of 10 mg every 2 weeks.

The dose-response curve suggests that 40 mg every 2 weeks may be the dose of maximal effect. The curve of the dose-response plot not only stops rising beyond this dose, it actually appears to decline, suggesting that higher doses may be somewhat detrimental to therapeutic effect (as seen with oral quetiapine (Arvanitis and Miller 1997) and oral risperidone (Ezewuzie and Taylor 2006)). The data at this end of the response curve, however, are mean doses, rather than fixed doses, and therefore contain a range of doses. The apparent decline in the curve in this dose range may be an artefact of ever-increasing doses for treatment-resistant patients (who were unlikely to respond to any dose). However, doses in the naturalistic or flexible dose studies rarely exceeded 60 mg every 2 weeks, suggesting that levels of treatment resistance were low. The study by Cookson (1987), which used the highest doses, included only treatment-responsive patients. Adding results from this study to the graph produces a marked flattening of the curve.

When only the fixed dose, relapse prevention studies were included, the curve shows no plateau, but stops at 26.66 mg every 2 weeks, thus limiting the ability to draw conclusions about higher doses and their possible effect.

Many of the studies were from times where additional antipsychotic use was a common practice. However, three quarters of the total sample came from studies where additional antipsychotics were not permitted. This allows reasonable estimation of dose-response for flupentixol decanoate without the confounding effects of other antipsychotic use. When including only these single-drug studies, the dose-response curve retains its S shape with a plateau around 30 mg every 2 weeks.

Studies in which the outcome was dropout for any reason were included, because often poor clinical effect is a reason for stopping medication (Lieberman et al. 2005; Taylor et al. 2006). The numbers of participants in these studies were small and did not alter the shape of the dose-response curve.

### Dose ranges used in the literature

Doses reported in the studies ranged from 1.7 mg per week to 800 mg every 2 weeks. We found evidence that a dose as low as 10 mg every 2 weeks was effective at preventing relapses in the medium term, and no evidence to support use of doses even approaching the highest UK recommended dose (400 mg/week).

The effective doses found here are consistent with the findings from other dose comparison RCTs. In their meta-analysis of relapse prevention studies, Uchida et al. (2011) find that low doses of antipsychotics [0.5–1 times the defined daily dose (DDD)] were as effective as standard doses (1 DDD or greater), whereas very low doses (less than 0.5 DDD) were inferior. The DDD for intramuscular flupentixol decanoate is 4 mg/day, equating to 56 mg every 2 weeks. Our results suggest that a dose of 0.35–0.7 DDDs is optimally effective at preventing relapses. As defined by the World Health Organisation (2018), the DDD is “the assumed average maintenance dose per day for a drug used for its main indication in adults”, not necessarily the recommended or most commonly prescribed dose.

In the UK and Ireland, flupentixol decanoate is marketed as Depixol® injection, and specifies a licensed dose range of 50 mg every 4 weeks up to a maximum of 400 mg every week for the maintenance treatment of schizophrenia. Our review suggests that doses beyond 40 mg every 2 weeks are unlikely to produce further beneficial effects for most patients. In other countries, e.g. Australia, Canada, France and New Zealand, flupentixol decanoate is marketed as Fluanxol® injection. The recommended dose range as stated in the product labels is 20–40 mg every 2 to 4 weeks in Australia and New Zealand, 20–40 mg every 2 to 3 weeks in Canada and 20–80 mg every 2 weeks in France. The Canadian product label also states that doses above 80 mg are rarely necessary but may be considered for individual patients. It is hard to explain why the dose ranges should differ so greatly between two versions of the same active ingredient produced by the same pharmaceutical company.

### Lowest effective dose

The study by Kistrup et al. (1991) demonstrated that for just under half of the studied patients, the lowest effective dose was between 20 and 40 mg every 2 weeks. For a further 30%, doses up to 80 mg every 2 weeks were sufficient, and only a quarter of patients apparently needed doses above 80 mg every 2 weeks.

The three dose reduction studies (Agrup-Andersson et al. 1974; Cookson 1987; Johnson et al. 1987) found higher relapse rates for previously stable patients who had their dose reduced by half compared with those who remained on their stable dose. The doses these patients had been stabilised on differed in each study, with one study only including patients stabilised on doses lower than 40 mg every 2 weeks and one study including only patients who had responded to doses above 100 mg every 2 weeks but not to lower doses. The third study, which allowed flexible dosing between 10 and 60 mg every 2 weeks prior to dose reduction, was the only one that found no statistically significant difference in relapse rates. These varied findings perhaps reflect the large differences in PK characteristics between individuals. Increased rates of relapse on dose reduction may also reflect dopamine supersensitivity psychosis (Chouinard et al. 1978).

In receptor occupancy studies, therapeutic doses of oral flupentixol led to D2-RO between 50 and 70%, with a corresponding ED_50_ of 0.68 ng/ml. No studies investigated D2-ROs for patients treated with flupentixol decanoate, and therefore, we cannot draw conclusions about the lowest dose of flupentixol decanoate that would lead to therapeutic D2-ROs.

### Maximum effective dose

None of the included studies were designed to identify a dose beyond which no further therapeutic response would be seen. The study that used the highest doses of flupentixol decanoate (Cookson 1987) did so for a subset of patients who responded only to dose increases beyond 100 mg every 2 weeks, when lower doses had been ineffective. Relapses were more frequent in those subjects who then had their dose reduced by half. This is perhaps the most compelling evidence that higher doses may be required in some individuals.

The highest dose administered in fixed dose studies was 26.66 mg every 2 weeks and producing a treatment success rate of 95% over 6 months in a relapse prevention study and 64% in an acute treatment study.

The receptor binding studies used oral flupentixol and did not find doses or serum levels at which saturation or near saturation of dopamine receptors occurs. Thus, a plateau of effect cannot be predicted from these studies.

### Dosing interval

No study investigated the effect of dosing interval on treatment failure. Treatment success rates were similar when grouped according to injection interval. The pharmacokinetic data suggests that two weekly injections provide the highest trough plasma level per dose administered and less fluctuation of serum levels within an individual. The apparent half-life of 17 days would also support an injection interval of 2 weeks. Only one study (Pach et al. 1998) specifically allowed an injection interval of 1 week, but it is unclear how many patients received their injection weekly. The time to maximal plasma concentration is 7 days, suggesting that weekly dosing is unnecessary unless a large volume of injection is to be given. Injection intervals of more than 4 weeks were not studied.

### Tolerability

One to two thirds of patients in the included studies experienced EPSE, although the exact number was dependent on the dose they received—severity of EPSE and incidence of tardive dyskinesia reduced with lower doses. However, doses below 10 mg every 2 weeks were not studied with respect to EPSE or therapeutic effect. The lack of a defined threshold dose for therapeutic effect makes it impossible to say with any certainty whether EPSE are always present in therapeutic use, that is, an inevitable consequence of therapeutic dosing.

Flupentixol has occasionally been described as a partial atypical antipsychotic due to its action on serotonin 5-HT_2A_ and 5-HT_2C_ receptors (Arnt 1998; Glaser et al. 1998), and it has long been suggested that typical drugs can be made essentially atypical by careful dosing (Kapur et al. 1996). Another criterion of atypicality is the lower incidence of EPSE at therapeutic doses. The atypical antipsychotics olanzapine and risperidone do not usually cause EPSE at their threshold doses and infrequently cause EPSE at their dose of near-maximal effect (Davis and Chen 2004; Bishara et al. 2013), but do produce significant EPSE at daily doses above 20 mg and 6 mg respectively. EPSEs were frequently reported when flupentixol was given at a dose of 40 mg every 2 weeks—our proposed near-maximal effect dose. The proportion of patients who were prescribed anticholinergic medicines at therapeutic doses was similar to that seen in haloperidol-treated patients in a recent head-to-head comparison study with paliperidone (McEvoy et al. 2014). So, even at the doses suggested here, flupentixol remains effectively a typical antipsychotic.

### Optimal dosing and recommendation for clinical practice

The optimal dose is the dose of drug that will produce the desired effect with the least likelihood of undesirable effects. The only study that aimed to find the optimal dose of flupentixol decanoate in the maintenance treatment of schizophrenia found that individualised doses between 5 mg and 40 mg every 1 to 4 weeks was the most successful (Pach et al. 1998). In this review, we found high success rates across the studied dose range. Relapse prevention for a chronic illness such as schizophrenia is desirable over a long period of time, and psychotic exacerbations may not be seen until over 12 months after a dose reduction (Marder et al. 1987). The relatively short period of 6 months may therefore not be long enough to estimate the dose that will prevent relapses in the longer term, although most of our included studies were longer than 6 months.

We found that rates of EPSE did not correlate with dose, but these were only reported in studies with mean doses of 40.7 mg every 2 weeks or less. Even at these doses, EPSEs were seen frequently. From this, we estimate the optimal dose of flupentixol decanoate in the maintenance treatment of schizophrenia to be between 20 mg and 40 mg every 2 weeks. We acknowledge the relative inconvenience of two weekly dosing and suggest that injection intervals should be individually established.

The average apparent half-life of flupentixol decanoate is 17 days, leading to a time to steady state of around 2 months. Effects of initial dose and subsequent dose changes on plasma levels will therefore take 2 months to become fully apparent. This delay makes dose titration difficult, and clinicians may be tempted to increase doses before the drug has reached steady state. Stabilisation with the more flexible oral flupentixol in the acute psychotic state is therefore preferable because dose can be more closely related to effects.

When switching to the long-acting injectable preparation, we suggest a starting dose of 20 mg every 2 weeks, which may be increased after 8 weeks to 30 mg or 40 mg every 2 weeks if necessary. For some patients, a lower dose of 10 mg every 2 weeks may be sufficient, and other patients may require higher doses. Doses above 100 mg every 2 weeks should only be given in exceptional circumstances, if tolerability allows.

For patients currently established on doses above 100 mg every 2 weeks, a cautious down-titration of dose may be warranted to reduce risk of tardive dyskinesia. The reviewed studies suggest that halving the dose in a previously stable patient puts them at danger of deterioration. It would therefore be pragmatic to reduce patients’ doses in small decrements with sufficient time between decrements to monitor the effect.

### Strengths and limitations

This review expands on the work by Reed and Fanshawe (2011) as it contains additional data from German-language studies, non-RCTs and the study by Dencker and co-workers (1980) which used flupentixol palmitate. This provided us with over twice as many patients as the review from 2011. We standardised treatment success rates to 6 months to account for time as a factor influencing relapse.

The included RCTs were small, making it harder to generalise the results. The larger, non-comparator studies had other limitations: e.g. the study by Chiliza et al. (2016) was primarily a feasibility and acceptability study and included first-episode patients only, and the study by Shajahan et al. (2010) included mostly middle-aged, Caucasian patients.

By standardising treatment failure rates to 6 months, we risk underestimating relapse rates. The longest studies (Gottfries and Green 1974; Wistedt and Ranta 1983) had the highest treatment failure rates over the entire study period but did report on relapse and dropouts at 6 months. For seven studies, treatment failure rates needed to be calculated, assuming a linear relapse rate. The study by Kane et al. (2012) demonstrated a linear relapse rate over the first 6 months for patients on treatment with aripiprazole long-acting injection, with relapses becoming less frequent after 6 months, but continuing at a linear rate for placebo-treated patients. Similar time-to-relapse plots were seen in the MARS study (Pach et al. 1998) and in the study by Shajahan et al. (2010).

Using mean doses as single data points to plot the dose-response curve can prove problematic, as they would more accurately be displayed as horizontal lines. Data from the fixed dose studies generally fit the curve, but do not provide relapse data beyond a dose of 26.67 mg every 2 weeks.

## Conclusion

This review suggests that the optimal dose of flupentixol decanoate in the maintenance treatment of schizophrenia is likely to be between 20 and 40 mg every 2 weeks, although some patients may be able to remain well on lower doses or on dosing intervals of 3 or 4 weeks. We suggest that doses of flupentixol should be individually established in the range of 10 to 40 mg every 2 weeks according to response and tolerability. Given pharmacokinetic variability, some patients may benefit from higher doses, but this should be the exception. Patients already established on higher doses may deteriorate if their dose is drastically or too quickly reduced. Dose reductions should therefore only take place after careful consideration of risks. Our suggested dose range is more in line with the product specifications of Fluanxol®, the brand under which flupentixol decanoate is marketed in countries outside of the UK and Ireland. We recommend that the Depixol® product label be revised to closer resemble that of Fluanxol®, which is more consistent with the evidence, as demonstrated in this review.
